# Decreased Preoperative Serum AGR as a Diagnostic Marker of Poor Prognosis after Radical Surgery of Upper Urinary Tract and Bladder Cancers from a Pooled Analysis of 9,002 Patients

**DOI:** 10.1155/2022/6575605

**Published:** 2022-09-05

**Authors:** Xiaoyan Wang, Guodong Yang, Yumeng Chai, Zhouyue Li, Xuanyan Che, Yongqiang Wang, Liqing Yang, Zhongbao Zhou

**Affiliations:** ^1^Department of Urology, Beijing Tiantan Hospital, Capital Medical University, No. 119 South 4th Ring West Road, Fengtai District, Beijing 100070, China; ^2^Department of Urology, Tengzhou Central People's Hospital, Tengzhou 277500, China; ^3^Department of Neurology, The Affiliated Yantai Yuhuangding Hospital of Qingdao University, No. 20 East Yuhuangding Road, Yantai 264000, Shandong, China

## Abstract

A growing number of studies have regarded the preoperative serum albumin-to-globulin ratio (AGR) as a prognostic indicator of urothelial carcinoma (UC) following radical surgery. However, a pooled analysis of AGR's effect on UC prognosis was still insufficient. Up to January 2022, a systematic search was conducted using PubMed, Embase, Web of Science, and Cochrane Library. Stata SE software was applied in this study. The reviewers collected the hazard ratio (HR) with 95% confidence interval (CI) for overall survival (OS), cancer-specific survival (CSS), recurrence-free survival (RFS), progression-free survival (PFS), and metastasis-free survival (MFS). A total of 9,002 patients from 12 retrospective studies were included in this analysis. The results showed that preoperative serum AGR was significantly associated with the OS (HR = 1.85, 95%CI = 1.43 to 2.39), CSS (HR = 2.38, 95%CI = 1.69 to 3.34), RFS (HR = 1.64, 95%CI = 1.29 to 2.08), PFS (HR = 2.16, 95%CI = 1.43 to 3.27), and MFS (HR = 3.00, 95%CI = 1.63 to 5.53) of patients with UC following radical surgery. Sensitivity analysis indicated the stability of the results. Subgroup analysis revealed that preoperative low AGR was seen as a risk factor for OS (HR = 1.90, 95%CI = 1.34 to 2.69), CSS (HR = 2.13, 95%CI = 1.40 to 3.26), and RFS (HR = 1.60, 95%CI = 1.24 to 2.07) in upper tract urothelial carcinoma (UTUC), but it was only a risk factor for CSS (HR = 2.95, 95%CI = 1.14 to 7.60) in bladder cancer (BC). Besides, preoperative AGR cut − value ≤ 1.4 could not be deemed as a stable prognostic indicator for RFS (HR = 2.07, 95%CI = 0.71 to 6.04) in UC. However, the predictive ability of AGR cut − value > 1.4 was stable. All in all, preoperative low AGR was considered as a risk factor for UC. AGR level can be regarded as a prognostic indicator for OS, CSS, and RFS in UTUC but only for CSS in BC. AGR greater than 1.4 can be a great cut-off value for predicting the prognosis of UC patients with radical operation.

## 1. Introduction

The most common malignant tumor of urinary system is urothelial carcinoma (UC), which is classified into upper tract urothelial carcinoma (UTUC) and bladder cancer (BC) [1, 2]. UTUC is a very uncommon cancer which contributes up 5% to 10% of all urothelial malignant tumors [3]. The standard therapy for nonmetastatic UTUC is radical nephroureterectomy (RNU) with bladder cuff excision [4]. Nevertheless, the recurrence rate of UTUC after RNU is significant, particularly in individuals with advanced malignancies [5]. BC is one of the tenth most common cancers, and muscle-invasive bladder cancer (MIBC) accounts for about 25% of all BC cases [6]. For individuals with MIBC, the recommended treatment is radical cystectomy (RC) followed by extensive pelvic lymph node dissection [7]. The prognosis of patients with MIBC has greatly improved with advances in surgical technologies and chemotherapeutic medicines in recent years; although, the five-year survival rate remains much lower than that of patients with other genitourinary tumors [8]. Therefore, the early prediction is critical for guiding later chemotherapy and follow-up regimens.

Multiple postoperative markers, such as clinical stage, pathological grade, and lymphovascular infiltration, are currently utilized to predict prognosis. However, these characteristics are generally examined by pathological assessment, making it difficult to assess clinical outcomes preoperatively [9, 10]. Reliable preoperative predictive indicators are important because individuals at greater risk of tumorigenesis would benefit from preoperative chemotherapy and lymph node resection [11]. Preoperative regular laboratory blood analysis, as one of quickest, most accessible, and least expensive diagnostic testing, has so far been proved to predict the outcomes of UC patients receiving radical surgery [12–14]. For two key aspects of human serum proteins, albumin (ALB) and globulin (GLB) in assessing nutritional quality and predicting disease prognosis have been well documented [15].

Several research have been conducted during last several years on forecasting the outcomes of UC patients by preoperative serum albumin to globulin ratio (AGR); although, it was still debatable. There is an urgent need for clear data establishing the predictive relevance of AGR in UC. The purpose of this pooled analysis was to determine the effect of preoperative AGR on patients with UC following radical surgery.

## 2. Methods

### 2.1. Search Strategy and Eligibility

The Preferred Reporting Items for Systematic Reviews and Meta-Analyses (PRISMA) and Assessing the Methodological Quality of Systematic Reviews (AMSTAR) guidelines were used to conduct this pooled analysis [16, 17]. The protocol for this study was not published on any public websites. Until Jan 2022, multiple databases including PubMed, Embase, Web of Science, and Cochrane Library were searched. The search keywords were as follows: (“albumin-to-globulin ratio” or “albumin” or “globulin”) and (“urothelial carcinoma” or “upper tract urothelial carcinoma” or “bladder cancer” or “bladder urothelial carcinoma”).

### 2.2. Study Selection Criteria

The inclusion criteria are as follows: [1] population: patients diagnosed with urinary tract cancers including UTUC or BC had AGR pretreatment of radical surgery; [2] intervention: AGR pretreatment (low); [3] comparator: AGR pretreatment (high); [4] outcomes: prognostic indicators; and [5] study designs: clinical research. Non-English language reports, in vitro studies, case reports, brief reports, conference abstracts/posters, and reviews were all excluded. If there were differences, the team would discuss and solve them. The inclusion criteria and exclusion criteria followed the PICOS principle (Table 1) [18].

### 2.3. Data Extraction

The extracted data were as follows: publication time, country, patient numbers, study design, tumor type, surgery type, AGR value (high/low), AGR value selection, median follow-up, overall survival (OS), cancer-specific survival (CSS), recurrence-free survival (RFS), progression-free survival (PFS), and metastasis-free survival (MFS). We contacted the corresponding authors to obtain more information if some indicators could not be derived from the original manuscript. Hazard ratio (HR) with 95% confidence interval (CI) extracted from multivariate analyses was prioritized. If only Kaplan-Meier curves were available, the relevant data were extracted using Engauge Digitizer 4.1 to calculate HR and 95% CI [19, 20].

### 2.4. Quality Assessment

This study adopted the Newcastle-Ottawa Scale (NOS) to evaluate the quality of selected studies, including three items: selection (1-4 points), comparability (1-2 points), and exposure (1-3 points), with total scores ranging from 0 (lowest) to 9 (highest) [21]. Studies with seven scores or more would be classified into high-quality and enrolled in the pooled analysis.

### 2.5. Statistical Analysis

All statistical analyses were performed using Stata E software. The association of preoperative AGR and OS, CSS, RFS, PFS, and MFS was evaluated by combining HR with 95% CI. We used high preoperative AGR as a reference, and HR > 1 indicated a negative impact of low preoperative AGR on UC patients. If HR with 95% CI was reported for high AGR versus low AGR, then HR for low AGR versus high AGR group would be obtained by Kaplan-Meier curves. The *I*^2^ statistic was adopted to assess the heterogeneity among several studies. Heterogeneity in this study was high; so, we adopted the random-effect model to reduce the effect of heterogeneity. Subgroup analysis was used to explore the effect of different classification on clinical outcomes. Sensitivity analysis was performed to assess whether each study significantly affected the pooled HR. *P* < 0.05 was considered statistically significant.

## 3. Results

### 3.1. Study Characteristics

**Figure 1:**
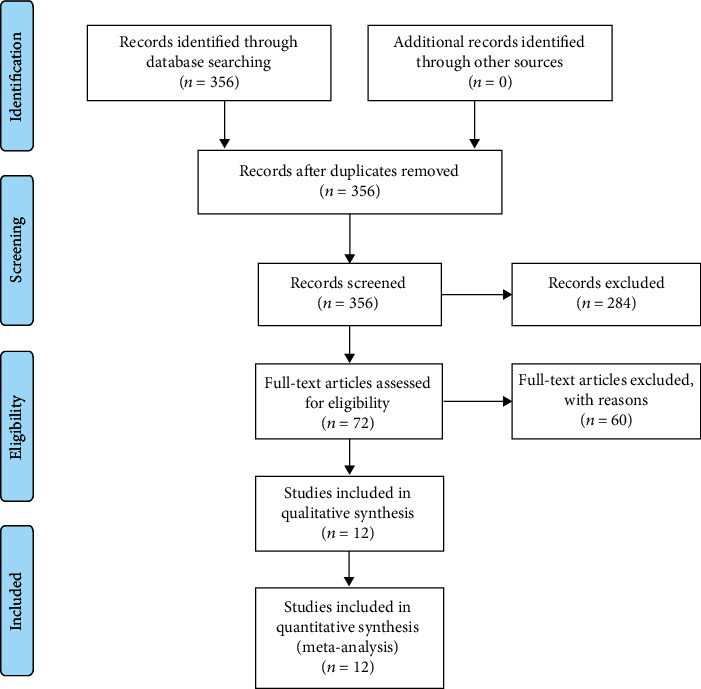
The PRISMA of selection process.

In the initial search, 356 studies were discovered. Based on the inclusion and exclusion criteria, 344 articles were excluded. Finally, 12 retrospective studies were included in the eventual analysis, including 9, 002 UC cases [22–33].  Table 2 summarizes the details of each study. The study screening process was shown in Figure 1. The quality of each study was rated as high quality by NOS tools (Table 3).

### 3.2. Overall Survival

**Figure 2:**
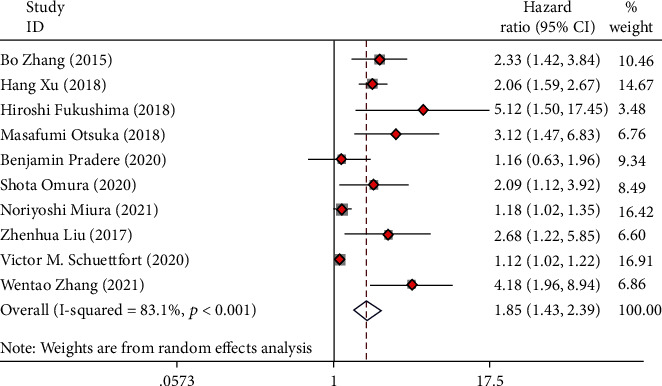
Forest plot of hazard ratio (HR) in UC patients for overall survival (OS).

Ten articles collecting 8,530 UC cases were involved in the research to analyze the relationship of preoperative AGR and OS. The forest plots reflected a HR of 1.85 (95% CI 1.43 to 2.39; *I*^2^ = 83.1%). The results revealed that low preoperative AGR was a risk factor for the OS of UC cases after radical operation (Figure 2).

### 3.3. Cancer-Specific Survival

**Figure 3:**
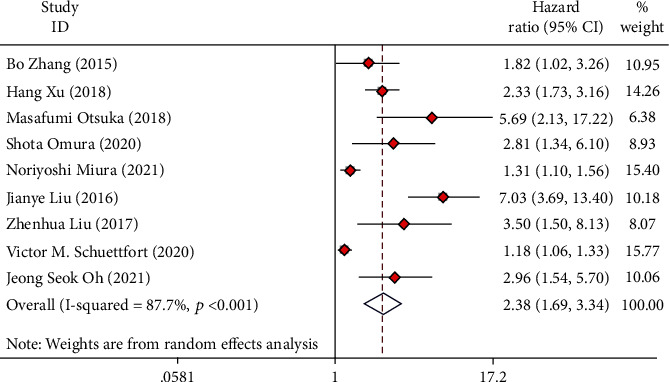
Forest plot of hazard ratio (HR) in UC patients for cancer-specific survival (CSS).

Nine articles collecting 8,590 UC cases were involved in the research to analyze the relationship of preoperative AGR and CSS. The forest plots reflected a HR of 2.38 (95% CI 1.69 to 3.34; *I*^2^ = 87.7%), which revealed that low preoperative AGR was a risk factor for the CSS of UC cases after radical operation (Figure 3).

### 3.4. Recurrence-Free Survival

**Figure 4:**
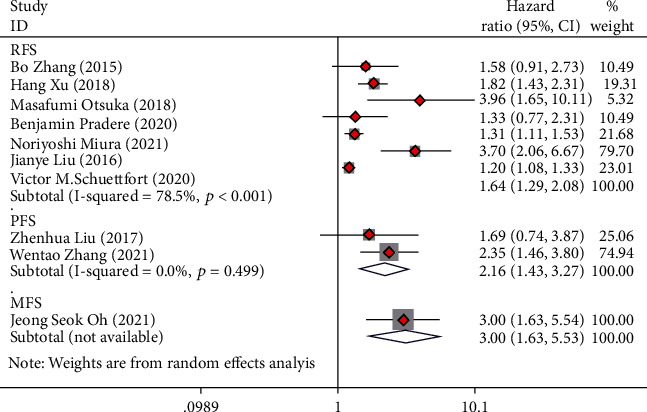
Forest plot of hazard ratios (HR) in UC patients for recurrence-free survival (RFS), progression-free survival (PFS), and metastasis free survival (MFS).

Seven articles collecting 8,039 UC cases were involved in the research to analyze the relationship of preoperative AGR and RFS. The forest plots reflected an HR of 1.64 (95% CI 1.29 to 2.08; *I*^2^ = 78.5%), which revealed that low preoperative AGR was a risk factor for the RFS of UC cases after radical operation (Figure 4).

### 3.5. Progression-Free Survival

Two articles collecting 316 UC cases were involved in the research to analyze the relationship of preoperative AGR and PFS. The forest plots reflected an HR of 2.16 and 95% CI of 1.43 to 3.27, which revealed that low preoperative AGR was a risk factor for the PFS of UC cases after radical operation (Figure 4).

### 3.6. Metastasis-Free Survival

One article collecting 176 UC cases was involved in the research for the relationship of preoperative AGR level and MFS. The forest plots reflected an HR of 3.00 and 95% CI of 1.63 to 5.53. The results revealed that low preoperative AGR was a risk factor for the MFS of UC cases after radical operation (Figure 4).

### 3.7. Subgroup Analysis Based on Tumor Type

**Figure 5:**
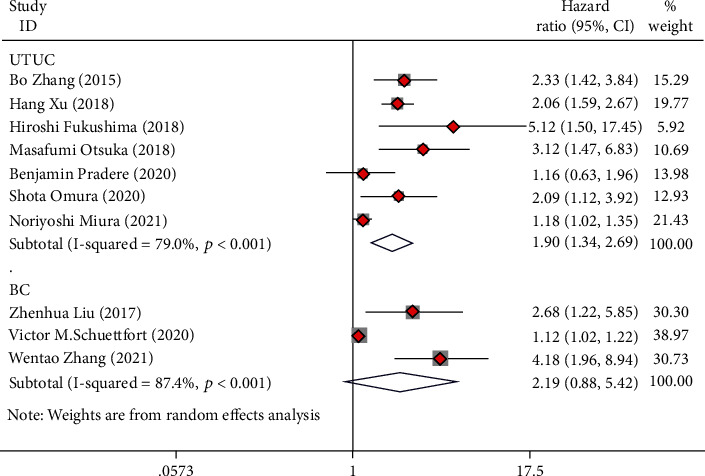
Forest plot of hazard ratios (HR) for overall survival (OS) according to tumor type including UTUC and BC.

**Figure 6:**
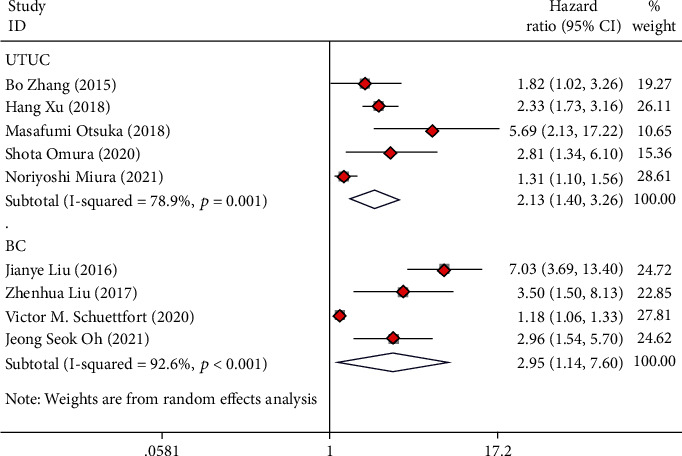
Forest plot of hazard ratios (HR) for cancer-specific survival (CSS) according to tumor type including UTUC and BC.

**Figure 7:**
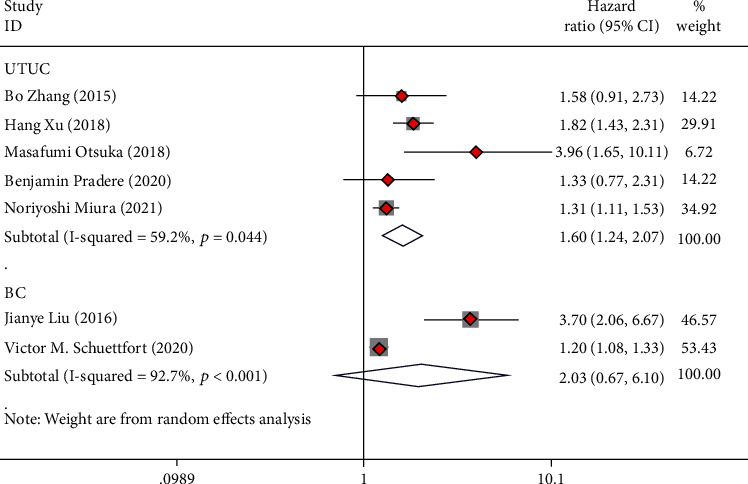
Forest plot of hazard ratios (HR) for recurrence-free survival (RFS) according to tumor type including UTUC and BC.

Of all the included studies, seven studies involved patients with UTUC who underwent RNU, and five studies involved BC patients who underwent RC. The analysis revealed that low preoperative AGR was seen as a risk indicator for OS (HR = 1.90, 95%CI = 1.34 − 2.69), CSS (HR = 2.13, 95%CI = 1.40 − 3.26), and RFS (HR = 1.60, 95%CI = 1.24 − 2.07) in UTUC cases (Figures 5–7). Low preoperative AGR was a risk indicator for CSS (HR = 2.95, 95%CI = 1.14 − 7.60) in BC cases after radical operation but not for OS (HR = 2.19, 95%CI = 0.88 − 5.42) and RFS (HR = 2.03, 95%CI = 0.67 − 6.10) (Figures 5–7).

### 3.8. Subgroup Analysis Based on AGR Cut Value

**Figure 8:**
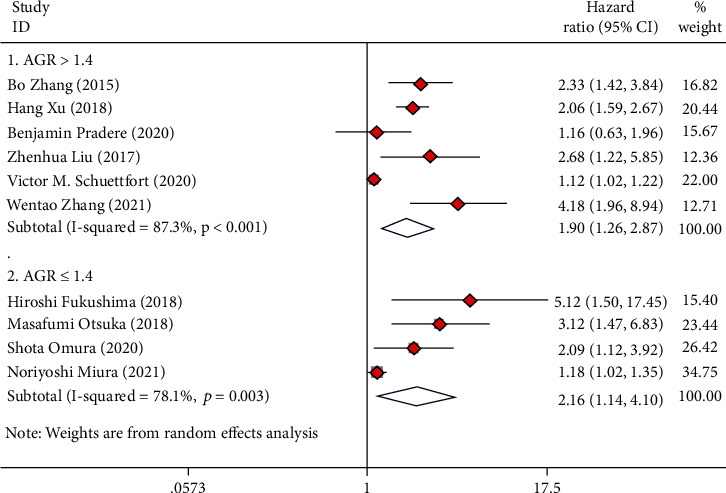
Forest plot of hazard ratios (HR) for overall survival (OS) according to AGR cut-value including ≤1.4 and >1.4.

**Figure 9:**
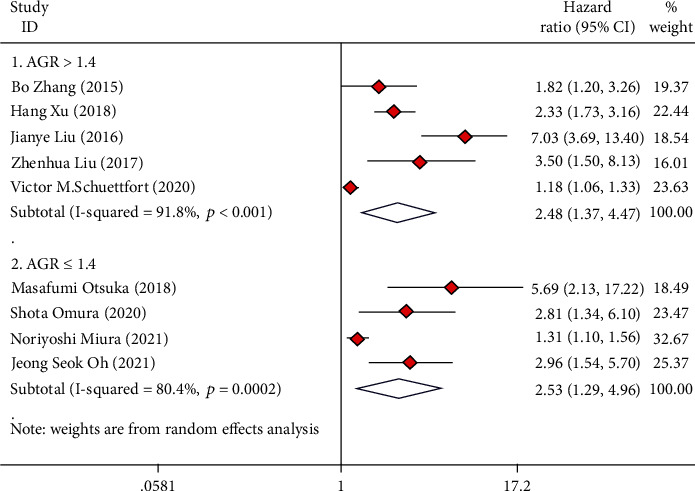
Forest plot of hazard ratios (HR) for cancer-specific survival (CSS) according to AGR cut-value including ≤1.4 and >1.4.

**Figure 10:**
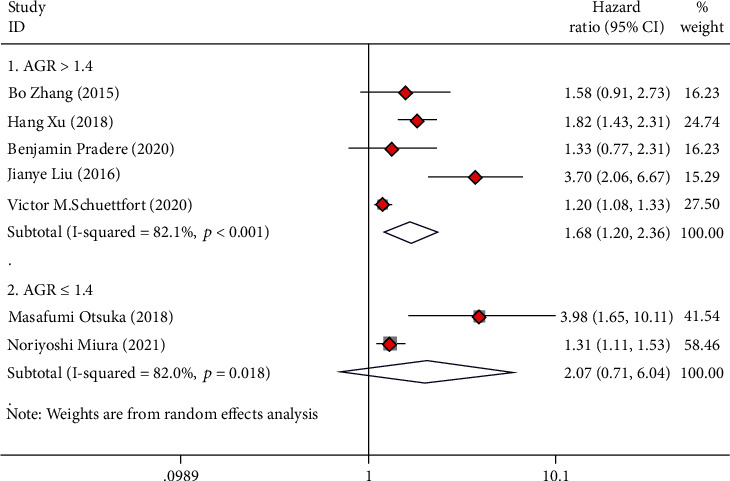
Forest plot of hazard ratios (HR) for recurrence-free survival (RFS) according to AGR cut-value including ≤1.4 and >1.4.

Among all the included studies, seven studies reported an optimal cutoff value of AGR greater than 1.4, and five studies reported an optimal cutoff value of AGR less than or equal to 1.4. The analysis revealed that the cut-off value of AGR greater than 1.4 can well predict OS (HR = 1.90, 95%CI = 1.26 − 2.87), CSS (HR = 2.48, 95%CI = 1.37 − 4.47), and RFS (HR = 1.68, 95%CI = 1.20 − 2.36) in UC cases after radical operation (Figures 8–10). The cut-off value of AGR less than or equal to 1.4 can well predict OS (HR = 2.16, 95%CI = 1.14 − 4.10) and CSS (HR = 2.53, 95%CI = 1.29 − 4.96) in UC cases after radical operation but not for RFS (HR = 2.07, 95%CI = 0.71 − 6.04) (Figures 8–10).

### 3.9. Sensitivity Analysis

**Figure 11:**
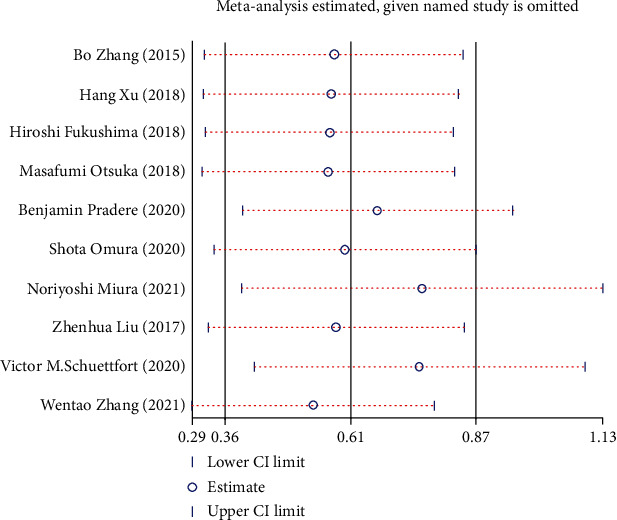
Sensitivity analysis for overall survival (OS) in UC patients.

**Figure 12:**
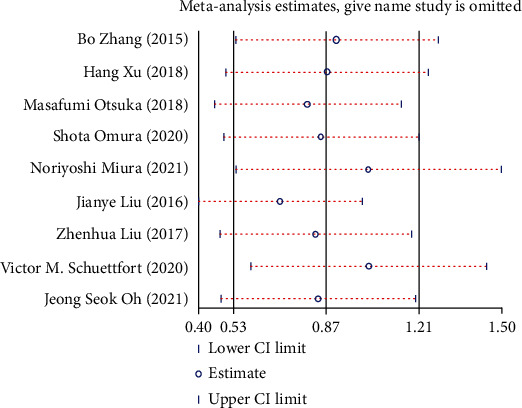
Sensitivity analysis for cancer-specific survival (CSS) in UC patients.

**Figure 13:**
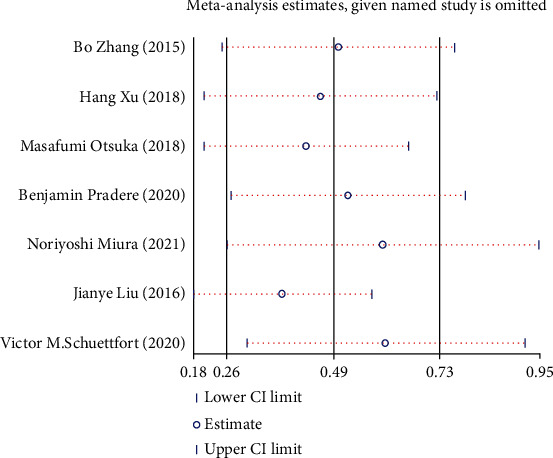
Sensitivity analysis for recurrence-free survival (RFS) in UC patients.

Sensitivity analysis was performed to assess whether each study significantly affected the pooled HR. The sensitivity analysis indicated that a single study could not significantly alter the pooled results of OS (Figure 11), CSS (Figure 12), and RFS (Figure 13) in UC cases after radical operation.

## 4. Discussion

This pooled analysis was conducted to investigate the prognostic value of preoperative AGR in UC cases after radical operation. A total of 9,002 patients from 12 eligible retrospective studies were included [22–33]. The results indicated that preoperative AGR was significantly related with the OS, CSS, RFS, PFS, and MFS of UC patients. Sensitivity analysis showed the stability of these results. Subgroup analysis revealed that a low preoperative AGR was seen as a risk indicator for the OS, CSS, and RFS of UTUC cases, but it was only a risk indicator for the CSS of BC cases. Moreover, the cut-off value of AGR greater than 1.4 can well predict OS, CSS, and RFS in UC cases after radical operation. The cut-off value of AGR less than or equal to 1.4 can well predict OS and CSS in UC cases after radical operation, but not for RFS.

Further study was needed to determine the relationship between decreased AGR and bad prognosis in patients with cancers. Nevertheless, existing data showed that lower diet, or hypoalbuminemia, was a risk factor for some cancers [13, 34]. The primary serum protein constituents are albumin and globulin, which are usually tested prior to surgery [35]. Albumin is commonly utilized in people with cancer to assess nutritional quality and systemic inflammation [36, 37]. The research has shown that lower serum albumin level was an independent prognostic factor of long-term survival in a variety of cancers [38]. Inflammatory process caused by serum globulins was essential for tumor growth, immune evasion, and spreading [34]. He et al. reported that serum globulins released by cancer tissues enhanced tumor formation, immunosuppressive, and cancer cell metastasis [34]. Furthermore, Laursen et al. discovered that serum albumin might modulate the capabilities of autocrine growth regulatory factors, which would really affect tumor growth [39]. Multiple studies have confirmed that serum albumin could reliably predict poorer oncologic results in UTUC patients [14, 38].

Therefore, a lower AGR can more sensitively estimate the extent of poor nutritional status and cancer growth than that of any parameter separately and may contribute as a directly important diagnostic indicator [22]. Multiple systemic inflammation indicators, such as neutrophil-to-lymphocyte ratio and lymphocyte-to-monocyte ratio, have already been created and widely studied in area of cancer, depending on a similar principle as AGR [40, 41]. Many studies have been performed in the last ten years to investigate the efficacy and specificity of preoperative AGR as a predicted prognostic marker for various malignancies [42]. Lower AGR was a potential risk factor for incidence of cancer and disease deaths in a normal monitoring community in both the short and long terms [43]. According to a meta-analysis of preoperative AGR and clinical malignancies, lower preoperative AGR was associated with the poorer OS, PFS, and disease-free survival [42]. Notably, the predictive effect of AGR remained independent of AGR cut-off values and cancer type, despite AGR cut-off ratios varied, varying between 0.9 and 1.93 for various malignancies [22]. The AGR cut-off ratio among UTUC subjects, on the other hand, was nearly consistent, varying between 1.4 and 1.45 [42].

This study reported that perioperative AGR can forecast poorer RFS, CSS, and OS before undergoing radical surgery. Despite growing evidence to the contrary, neoadjuvant chemotherapy was the acknowledged common treatment for MIBC but not for UTUC [44]. Although more advances in contemporary imaging methods such as computed tomography and magnetic resonance imaging, accurate staging of UTUC preoperatively was challenging. Because of the small size of tissue samples, preoperative UTUC assessment with histology was especially difficult [45]. Since difficulties in preoperative UTUC staging and histology classification, some individuals may be overtreated while others managed with RNU monotherapy may be undertreated [46]. Considering the loss of renal function accompanied with nephrectomy, a neoadjuvant treatment was an appealing alternative for individuals who were probably to need it and benefit [47]. As a result, reliable preoperative indicators which can be capable of identifying patients for neoadjuvant therapy were required [48]. Preoperative AGR may be a valuable indicator for decision support in individuals experiencing preoperative systemic treatment guidance, whereas the pooled analysis demonstrated a high connection between AGR and UC prognosis, and some limitations should be noted. Firstly, all the research used retrospective methods, which raised the possibility of bias. Secondly, dietary inadequacies, illnesses, drugs, and lifestyle can influence blood-based indicators, resulting in a bias. Thirdly, while the random-effect model took into account the variability of studies, the results must be carefully considered when using it. Finally, the majority of patients in our research came from a specific region of Asia, which may make it difficult to generalize the results.

## 5. Conclusions

Preoperative low AGR was considered as a risk factor for UC. AGR level can be regarded as a prognostic indicator for OS, CSS, and RFS in UTUC but only for CSS in BC. AGR greater than 1.4 can be a great cut-off value for predicting the prognosis of UC patients with radical operation.

## Figures and Tables

**Table 1:** Population, Intervention, Comparator, Outcomes, and Study Designs (PICOS) structure.

Items	Populations	Intervention	Comparator	Outcomes	Study designs
Inclusion criteria	Patients diagnosed with urinary tract cancers including upper urinary tract urothelial carcinoma and bladder urothelial cancer had AGR pretreatment of radical surgery.	AGR pretreatment (low)	AGR pretreatment (high)	Overall survival, cancer-specific survival, recurrence-free survival, progression-free survival, and metastasis-free survival.	Clinical research.
Exclusion criteria	Patients with urinary tract cancers undergoing nonradical surgery or palliative therapy or no AGR pretreatment.	Other	Other	Patient feelings; inadequate indicators.	Letters, comments, reviews, case reports, abstracts, and animal experiment.

AGR: albumin-to-globulin ratio.

**Table 2:** The characteristics of included studies.

Study	Study design	Country	Sample size	Tumor	Treatment	AGR value (high/low)	AGR value selection	Outcome	Median follow-up (months; range)
Zhang et al. [28]	Retrospective study	China	187	UTUC	Radical nephroureterectomy	1.45	ROC	OS; CSS	78 (32-92)
Xu et al. [27]	Retrospective study	China	620	UTUC	Radical nephroureterectomy	1.45	ROC	OS; CSS; RFS	50 (28-78)
Fukushima et al. [26]	Retrospective study	Japan	105	UTUC	Radical nephroureterectomy	1.24	ROC	OS	46 (22-83)
Otsuka et al. [25]	Retrospective study	Japan	124	UTUC	Radical nephroureterectomy	1.4	ROC	OS; CSS; RFS	55 (28-76)
Pradere et al. [24]	Retrospective study	Multicenter	172	UTUC	Radical nephroureterectomy	1.42	ROC	OS; RFS	26 (11-56)
Omura et al. [23]	Retrospective study	Japan	179	UTUC	Radical nephroureterectomy	1.25	ROC	OS; CSS	34 (17-63)
Miura et al. [22]	Retrospective study	Multicenter	2492	UTUC	Radical nephroureterectomy	1.4	ROC	OS; CSS; RFS	38 (NA)
Liu et al. [33]	Retrospective study	China	296	BC	Radical cystectomy	1.6	ROC	CSS; RFS	72 (49.75-115.50)
Liu et al. [32]	Retrospective study	China	189	BC	Radical cystectomy	1.55	ROC	OS; CSS; PFS	38 (1-90)
Victor et al. [31]	Retrospective study	Multicenter	4335	BC	Radical cystectomy	1.42	ROC	OS; CSS; RFS	31.5 (13.3-72.3)
Jeong Seok Oh et al. [30]	Retrospective study	Korea	176	BC	Radical cystectomy	1.32	ROC	CSS; MFS	32.4 (0.2–95.3)
Zhang et al. [29]	Retrospective study	China	127	BC	Radical cystectomy	1.55	ROC	OS; PFS	Until March 2018

UTUC: upper tract urothelial carcinoma; BC: bladder cancer; ROC: receiver operating curve; OS: overall survival; CSS: cancer-specific survival; RFS: recurrence-free survival; PFS: progression-free survival; MFS: metastasis-free survival; AGR:

albumin-to-globulin ratio.

**Table 3:** Quality assessment of the included studies.

Study	Selection				Comparability	Exposure			Score
	Definition adequate	Represent of cases	Selection of controls	Definition of controls		Ascertainment of exposure	Same method of ascertainment	Nonresponse rate	
Zhang et al. [28]	⨁	⨁	⨁	⨁	⨁	⨁	⨁	⨁	8
Xu et al. [27]	⨁	⨁	⨁	⨁	⨁⨁	⨁	⨁	⨁	9
Fukushima et al. [26]	⨁	⨁	⨁	⨁	⨁	⨁	⨁	◯	7
Otsuka et al. [25]	⨁	⨁	◯	⨁	⨁	⨁	⨁	⨁	7
Pradere et al. [24]	⨁	⨁	⨁	⨁	⨁⨁	⨁	⨁	⨁	9
Omura et al. [23]	⨁	⨁	⨁	⨁	⨁⨁	⨁	⨁	⨁	9
Miura et al. [22]	⨁	⨁	⨁	⨁	⨁⨁	◯	⨁	⨁	8
Liu et al. [33]	⨁	⨁	⨁	⨁	⨁⨁	⨁	⨁	⨁	9
Liu et al. [32]	⨁	⨁	⨁	⨁	⨁	⨁	⨁	⨁	8
Victor et al. [31]	⨁	◯	⨁	⨁	⨁⨁	⨁	⨁	⨁	8
Jeong Seok Oh et al. [30]	⨁	⨁	⨁	⨁	⨁⨁	⨁	⨁	◯	8
Zhang et al. [29]	⨁	⨁	⨁	⨁	⨁	⨁	⨁	⨁	8

## Data Availability

The datasets used and/or analyzed during the current study available from the corresponding authors on reasonable request.
